# Tumor Necrosis Factor-α Sensitizes Breast Cancer Cells to Natural Products with Proteasome-Inhibitory Activity Leading to Apoptosis

**DOI:** 10.1371/journal.pone.0113783

**Published:** 2014-11-24

**Authors:** Li Lu, Wenli Shi, Rahul R. Deshmukh, Jie Long, Xiaoli Cheng, Weidong Ji, Guohua Zeng, Xianliang Chen, Yajie Zhang, Q. Ping Dou

**Affiliations:** 1 Department of Pathophysiology, Guangzhou Medical University, Guangzhou, Guangdong Province, China; 2 Department of Pathology and Karmanos Cancer Institute, School of Medicine, Wayne State University, Detroit, Michigan, United States of America; 3 Department of Pathology, Guangzhou Medical University, Guangzhou, Guangdong Province, China; 4 Department of Urology, Minimally Invasive Surgery Center, The First Affiliated Hospital of Guangzhou Medical University, Guangdong Provincial Key Laboratory of Urology, Guangzhou, Guangdong Province, China; 5 Departments of Oncology and Pharmacology, School of Medicine, Wayne State University, Detroit, Michigan, United States of America; Swedish Medical Center, United States of America

## Abstract

The inflammatory microenvironment plays an important role in the process of tumor development. Tumor necrosis factor-α (TNF-α), a key pro-inflammatory cytokine, has a significant role in this process. Natural medicinal products such as Withaferin A (WA) and Celastrol (Cel) have shown anti-cancer and anti-inflammatory properties that can be attributed to multiple mechanisms including, but not limited to, apoptosis induction due to the inhibition of proteasomal activities. This study aimed to investigate the effects of TNF-α in combination with WA or Cel in vitro in MDA-MB-231 breast cancer cells. TNF-α, when combined with WA or Cel, activated caspase-3 and -9 and downregulated XIAP in a dose-dependent manner, leading to induction of apoptosis in MDA-MB-231 breast cancer cells. The combination also caused accumulation of the proteasomal target protein IκBα, resulting in inhibition of the nuclear translocation of nuclear factor-κB (NF-κB). Taken together, these results suggest that TNF-α could sensitize breast cancer cells MDA-MB-231 to WA and Cel, at least in part, through inhibiting the activation of NF-κB signaling, leading to XIAP inhibition with subsequent upregulation of caspase-3 and -9 activities. Thus, the anti-cancer activities of TNF-α are enhanced when combined with the natural proteasome inhibitors, WA or Cel.

## Introduction

Natural products have potential as anticancer therapies due to their anti-inflammatory and tumor-suppressing properties [1]. However, the mechanisms that regulate these properties are poorly understood. Withaferin A (WA), a natural product isolated from the Indian medicinal plant *Withania somnifera*, has shown anti-tumor, anti-angiogenic and radio-sensitizing activities in many cancer cell systems [2], [3]. Its anti-cancer activities have been demonstrated in breast [4], leukemia [5], prostate [6], [7] and melanoma [8] cancer cells. WA induces apoptosis in prostate cancer cells via Par-4 induction [7], inhibition of nuclear factor-κB (NF-κB) activation [3], covalent modification of the cysteine residue on vimentin [9], and inhibition of the chymotrypsin-like (CT-like) activity of the proteasome [6].

Celastrol (Cel), a tripterine compound isolated from a traditional Chinese medicinal plant *Trypterygium wilfordii Hook F*. (“Thunder of God Vine”) has shown therapeutic potential in chronic inflammatory disorders, such as lupus erythematosus [10], arthritis [11], Alzheimer's disease [12] and lateral sclerosis [13]. It also induces apoptosis in different types of cancer cell lines via inhibition of IκBα kinase [14], [15], proteasome [16], topoisomerase activity [17], heat shock protein [18] and VEGF receptor expression [19].

Inflammation plays a major role in the process of tumorigenesis. It has been shown that the inflammatory microenvironment is essential at different stages of tumor development. However, the direct link between inflammation and tumor development is yet to be identified [20], [21]. Tumor necrosis factor-α (TNF-α) is one of the major pro-inflammatory cytokines and paradoxically can be either a tumor promoter linking inflammation with carcinogenesis or a tumor inhibitor as it induces cancer cell death due to the sustained JNK activation. Thus, it can promote tumor cell proliferation, survival, migration and angiogenesis as well as being able to induce cancer cell death, making it a double-edged sword in cancer therapy. It is therefore important to find out how to selectively trigger the anti-tumor properties of TNF-α while avoiding or inhibiting its tumorigenic properties [20], [21], [22], [23].

Cellular responses to TNF-α are mediated to a large degree by a transcription factor called nuclear factor-κB (NF-κB) [22], [23]. Studies have shown that NF-κB protects most cells and tissues from apoptosis. Its anti-apoptotic activity results from transcriptional activation of a large number of anti-apoptotic proteins such as c-FLIP, Bcl-2, Bcl-XL, cIAP2, and A1/Bfl-2. When p65, one of the subunits of the NF-κB complex was inactivated in mice, enhanced apoptosis was observed [24]. Activation of NF-κB signaling due to TNF-α helps tumor cells to escape TNF-α-induced cytotoxicity [22], [23], [25].

Inhibitor of apoptosis (IAP) family proteins regulate apoptosis by endogenously inhibiting caspases. It should be noted that IAPs are over-expressed in various tumors [26], [27]. To date, eight members of the human IAP family including cIAP1, cIAP2 and XIAP have been reported. It has been demonstrated that XIAP binds and specifically inhibits caspase-3, -7, and -9 [26], [27], [28], [29] and it is believed that it plays a role in modulation of inflammatory signals via activation of NF-κB [30], although the mechanism by which XIAP mediates these effects under physiological conditions is not clear.

Based on the essential requirement for an inflammatory microenvironment in tumor formation, we investigated the effects of TNF-α on apoptosis in vitro in breast cancer MDA-MB-231 cells when combined with natural products with proteasome-inhibitory activities. We discovered that TNF-α, when combined with WA or Cel, effectively sensitized breast cancer MDA-MB-231 cells to TNF-α-mediated induction of apoptosis by targeting its effector signaling pathway, i.e. NF-κB. Due to their proteasome-inhibitory activities, WA and Cel impaired NF-κB signaling, contributing to their anti-inflammatory as well as anti-cancer activities. Furthermore, increased inhibition of XIAP resulting in apoptosis was observed in these breast cancer cells when treated with a combination of TNF-α and WA or Cel.

## Materials and Methods

### Reagents

WA and Cel were purchased from A. G. Scientific, Inc. (San Diego, CA, USA) and dissolved in DMSO (Sigma-Aldrich, St. Louis, MO, USA). 50 mM Stock solutions were prepared and aliquots were stored at −20°C for further use in the experiments. L15 (Leibovitz's L-15 with l-glutamine), penicillin, and streptomycin were purchased from Invitrogen (Carlsbad, CA, USA). Fetal bovine serum (FBS) was obtained from Gibco Technology (Brasil, USA). Bisbenzimide, methylene blue, 3-[4, 5-dimethyltiazol-2-yl]-2.5-diphenyl-tetrazolium bromide (MTT), RNaseA, reverse transcription and real-time quantitative polymerase chain reaction (RT-qPCR) kits were purchased from Promega (San Luis Obispo, CA, USA). Ac-Asp-Glu-Val-Asp-AMC and Z-Gly-Gly-Leu-AMC, the substrates for caspase-3 and the chymotrypsin (CT)-like activities respectively were obtained from Calbiochem Inc. (San Diego, CA). Mouse monoclonal antibodies against human poly (ADP-ribose) polymerase (PARP), mouse monoclonal antibodies against ubiquitin (P4D1), rabbit polyclonal antibody against IκBα (C-15), and goat polyclonal antibodies against β-actin (C-11), caspase-3, caspase-9, XIAP, IAP1/2 as well as secondary antibodies were purchased from Santa Cruz Biotechnology (Santa Cruz, California, U.S.A.).

### Cell culture and whole cell extract preparation

Human breast cancer MDA-MB-231 cells were obtained from the American Type Culture Collection (Manassas, VA, USA) and grown in L15 supplemented with 10% FBS and 100 U/ml penicillin as well as 100 µg/ml streptomycin. All cells were maintained at 37°C with 5% CO_2_. Whole cell extracts were prepared as described previously [6], [31]. Briefly, cells were harvested, washed with phosphate buffered saline (PBS) and homogenized for 30 min at 4°C in a lysis buffer (50 mM Tris-HCl, pH 7.5, 150 mM NaCl, 0.5% NP-40, 0.5 mM phenylmethylsulfonyl fluoride, and 0.5 mM dithiothreitol). The lysates were immediately centrifuged at 12,000 rpm for 12 min at 4°C and the supernatants were collected as whole cell extracts. Protein concentration was determined using a bicinchoninic acid protein assay (Pierce Biotechnology, Rockford, IL, USA) and bovine serum albumin (BSA) was used as standards.

### MTT assay

MTT assays were performed in 96-well plates. Briefly, 3.5×10^3^ MDA-MB-231 cells were seeded per well and incubated overnight. The cells were treated with either WA (0.625–20 µM) or Cel (0.25–8 µM) alone or in combination with TNF-α (10 ng/ml) for 48 hours. DMSO treated cells were taken as control. Inhibition of cell proliferation was determined using the MTT assay as described in detail elsewhere [31]. Absorbance was measured using a Wallac Victor3 multilabel counter at 540 nm, and cell viability was expressed relative to the DMSO treated control cells. In individual experiments, each treatment condition was set up in quadruplicate, and each experiment was repeated 1 to 5 times independently. IC_50_ was calculated by Origin software.

### Colony formation assays

Colony formation assays were performed in 6-well plates. Briefly, 350 MDA-MB-231 cells were seeded per well. After overnight incubation, cells were treated with WA (1, 2 µM) or Cel (1, 1.5 µM) either alone or in combination with TNF-α (10 ng/ml) for 24 hours. The drugs were subsequently removed and the cells were kept in culture by refreshing growth medium every two days, during which time the surviving cells produced a colony of proliferating cells. On day 10, colonies were washed with ice-cold PBS and stained with 1% methylene blue solution in methanol for 4 hours. Colony forming efficiency was calculated and images were taken. Each treatment condition was set up in triplicate in individual experiments.

### Cell gap closure migration assays (scratch wound-healing assay)

MDA-MB-231 cells were seeded in 6-well plates at a density of 5×10^5^/well. When cells were approximately 80% confluent, the cell monolayer was scratched with pipette tips and washed once with PBS. WA (0.5, 1 µM) or Cel (0.5, 1 µM) either alone or in combination with TNF-α (diluted in the culture medium immediately before addition) were added to each well at the desired final concentrations and incubated for 24 hours. Migration of cells was observed with an inverted microscope and photographs were taken within the same field of vision at 0 hour and 24 hours. The actual migration speed was calculated by Image-Pro plus.

### Cell death detection assays

MDA-MB-231 cells were seeded in 6-well plates at 3.5×10^5^ cells per well. After overnight incubation, cells were treated (in triplicate) with a final concentration of WA (1, 2 µM) or Cel (0.5, 1 µM) either alone or in combination with TNF-α (10 ng/ml) for 24 hours. Cells were then harvested and washed with ice-cold PBS and resuspended with the binding buffer, followed by incubation with Annexin V-FITC (solution in 25 mM HEPES, 140 mM NaCl, 1 mM EDTA, pH 7.4, 0.1% BSA) (Jiamei Biotechnology, Beijing, China) for 15 min and Propidium Iodide (PI) (1.5 mM solution in deionized water) (Jiamei Biotechnology, Beijing, China) for additional 15 min at 4°C in darkness. The stained cells were analyzed by flow cytometry (Ex/Em = 488 nm/530 nm) within 30 min.

### Caspase-3 activity, chymotrypsin (CT)-like activity assay and Western blotting analysis

Caspase-3 activity was determined by measuring the release of the AMC groups from a caspase-3 specific substrate Ac-Asp-Glu-Val-Asp-AMC. MDA-MB-231 Cells (5.0–8.0×10^5^/well) were plated and after overnight incubation were treated with the indicated concentrations of WA or Cel either alone or in combination with TNF-α (10 ng/ml) for the indicated time periods, after which, whole cell extracts were prepared. Cell extracts (25 mg) were then incubated in a 96-well plate in 100 µl of assay buffer (50 mM Tris-HCl, pH 7.5) with 40 µM Ac-Asp-Glu-Val-Asp-AMC. The reaction mixture was incubated at 37°C for 2 hours and the hydrolyzed fluorescent AMC groups were quantified as previously described [6], [31].

Proteasomal CT-like activities were determined by measuring the release of the AMC groups from a CT-like specific substrate Z-Gly-Gly-Leu-AMC. MDA-MB-231 Cells (5.0–8.0×10^5^/well) were plated in a 96-well plate and treated with WA or Cel at the desired final concentrations either alone or in combination of TNF-α (10 ng/ml) for 8 hours or for the indicated time periods and incubated with Z-Gly-Gly-Leu-AMC (at 40 µM) for an additional 2 hours. The production of hydrolyzed AMC group was measured as previously described [6], [31].

For Western blotting analysis, cells (5.0–8.0×10^5^/ml) were plated in 60 mm dishes and treated with the desired final concentration of WA or Cel either alone or in combination with TNF-α for different time periods, followed by preparation of whole cell extracts. Western blotting assays were performed using an enhanced chemiluminescence reagent as previously described [6], [31].

### Preparation of nuclear extracts for NF-κBp65 and Western blotting analysis

Nuclear extracts were prepared according as described previously [32]. Briefly, 1×10^7^cells of each group were washed with cold PBS and suspended in 0.4 ml hypotonic lysis buffer containing protease inhibitors for 30 min. The cells were then lysed with 10% Nonidet P-40.

### Immunofluorescence microscopy

Fluorescent immunostaining was performed as described previously [33]. Briefly, 1×10^5^/well cells were treated with WA or Cel at the desired concentration either alone or in combination with TNF-α (10 ng/ml) for 1.5 hours. Cells were incubated with 1∶100 NF-κBp65 mouse antibody. Slides were reprobed with 1∶150 Alexa Fluor 555-conjugated goat anti-mouse IgG. Cell nuclei were counterstained with Hoechst 33258. Fluorescence images were observed under a Zeiss microscope.

### Reverse transcription and RT-qPCR

Cells (5×10^6^) were pretreated with different doses of WA (1, 2 µM) and Cel (0.5, 1 µM) either alone or in combination with TNF-α (10 ng/ml) for 12 hours at 37°C. Total RNA was extracted using Trizol reagent according to the manufacturer's instructions. The genomic DNA was digested with RNase-free DNaseI and the concentration of RNA was measured. Single-stranded cDNA was generated with random hexamer primers using the Prime Script RT Master Mix first strand synthesis system for RT-qPCR. After the reverse transcription, RT-qPCR was performed with the SYBR Premix Ex Taq II (Tli RNaseH Plus). An 18s primer set was used as an internal control. The PCR primers used were as follows:

XIAP-F, ACATGGCTGTCAAGAAGG-AGAT,

XIAP-R, ACTGCAGCCTCGAACTTCTG, 180 bp.

cIAP1/2-F, TTCCGTGGCTCTTATTCAAACT,

cIAP1/2-R, GCACAGTGGTAGGAACTTCTCAT, 96 bp.

NF-κBp65-F, CTCCGAGACTTTCGAGGAAATAC

NF-κBp65-R, GCCATTGTAGTTGGTAGCCTTCA, 135 bp.

18sr-F, CCTGGATACCGCAGCTAGGA,

18sr-R, GCGGCGCAATACGAATGCCCC, 112 bp.

### Gene silencing using siRNA

The custom designed small interfering RNA (*si*RNA) for NF-κBP65 and non silencing control *si*RNA were purchased from Santa Cruz Biotechnology, Inc. (Santa Cruz, CA, USA). For transient expression, cells were transfected using Lipofectamine 2000 reagent. After incubating cells for 6 hours, the lipid and siRNA complex was removed and fresh growth medium and TNF-α (10 ng/ml) were added. Cells of other groups were respectively treated with the desired final concentration of WA or Cel in combination with TNF-α for 24 or 48 hours. Specific mRNA and protein levels were determined by RT-qPCR and Western blotting analysis.

### Statistical analysis

Data are expressed as mean ± SD. Student's *t*-test was applied to evaluate the differences between treated groups and controls. Data from multiple groups were analyzed by one-way ANOVA, followed by the Tukey-Kramer multiple comparison test. For all tests, a *P*-value<0.05 was considered to reflect a statistically significant difference between groups.

## Results

### TNF-α sensitized breast cancer cells to WA and Cel resulting in induction of cell death

**Figure 1:**
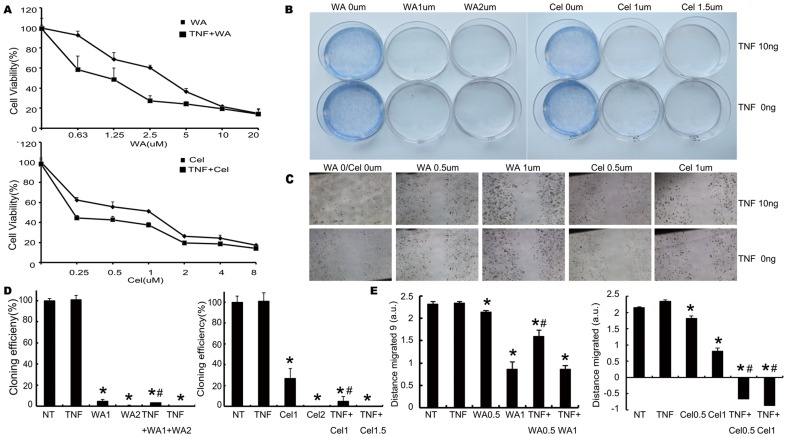
WA and Cel sensitize MDA-MB-231 cells to TNF-α resulting in inhibition of cell proliferation. (A) Concentration dependent effect of WA or Cel with or without TNF-α on cell viability. MDA-MB-231 cells were treated with different concentrations of WA (upper) or Cel (lower) with or without TNF-α (10 ng/ml) for 48 hours, followed by measurement of cell viability by MTT assay. IC_50_ was calculated by Origin. (B) Colony formation assays of cells treated with WA or Cel with or without TNF-α. WA (left) or Cel (right) at shown concentrations with or without TNF-α (10 ng/ml) were added to the cells for 24 hours. The medium was subsequently removed and the cells were maintained in culture for a further 10 days. (C) Scratch wound-healing assay for cells treated with WA or Cel with or without TNF-α at shown concentrations. Photographs were taken at 24 hours after treatment and scratching of the well. (D) Colony forming efficiency was calculated. (E) Actual migration speed was calculated by Image-Pro plus. Data are shown as mean ± SD of three experiments. (*P<0.05, versus the untreated group. #P<0.05, WA/Cel treated versus WA/Cel+TNF-α).

It has been reported that normal serum TNF-α levels are in the pg/ml range but they are approximately 20–25% higher in breast cancer tissue as compared to normal tissue [34], [35], [36]. In order to mimic the inflammatory conditions in cancer tissues, 10 ng/ml of TNF-α was used for each experiment in this study. MTT assays were performed to investigate the effects of combining TNF-α with WA or Cel on MDA-MB-231 cancer cell proliferation. TNF-α treatment itself did not have any effect on the cell proliferation, but when combined with WA (0.625–2.5 µM) or Cel (0.25–2.0 µM), it significantly enhanced the natural product's ability to inhibit MDA-MB-231 cell proliferation (p<0.05). For example, TNF-α when combined with WA at the dose of 0.625 µM decreased cell proliferation to 60% from 93% (WA alone). TNF-α plus WA/Cel significantly inhibited the cell viability of MDA-MB-231 cells in a concentration-dependent manner (Fig. 1A). The IC_50_ value of WA with TNF-α decreased to 2.63 µM compared to 7.23 µM (WA alone). Similarly, the IC_50_ of Cel alone (2 µM) decreased when combined with TNF-α (0.58 µM) (Fig. 1A). To further validate these results, we performed colony formation assays and observed similar inhibitory effects on cell proliferation with the combination of TNF-α with WA or Cel. TNF-α itself had no effect on colony formation but WA or Cel alone inhibited colony formation in a dose-dependent manner, which is consistent with the results of the MTT assays. The inhibition of colony formation was enhanced by the combination of TNF-α with WA or Cel compared to WA or Cel treatments alone (Fig. 1B, D).

The cell scratch wound healing assay is a simple and inexpensive method of evaluating cell migration ability. This technique can be used to measure the invasion and metastasis abilities of adherent tumor cells. We chose two doses of WA (0.5, 1 µM) and Cel (0.5, 1 µM), respectively, based on the previous results of MTT and colony formation assays. TNF-α combination with WA or Cel (0.5, 1 µM) significantly inhibited the cell migration in a dose-dependent manner (Fig. 1C, E).

### TNF-α sensitized breast cancer cells to WA and Cel resulting in induction of apoptosis

**Figure 2:**
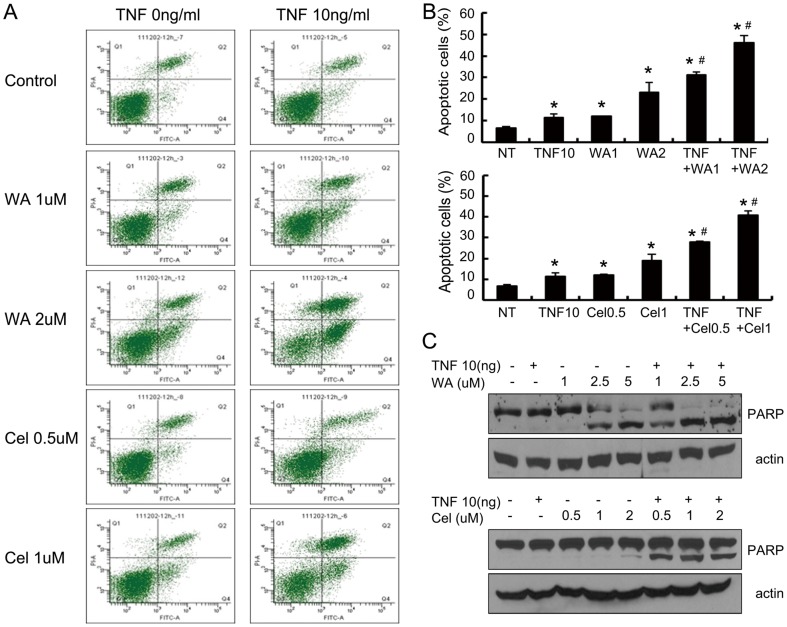
WA or Cel sensitize MDA-MB-231 cells to TNF-α-induced apoptosis. (A, B) Cells were treated with different concentrations of WA or Cel with (right) or without (left) TNF-α (10 ng/ml) for 48 hours, followed by measurement of cell viability by flow cytometry. (C) Cell death inducing abilities of WA and TNF-α or Cel and TNF-α combination in a dose responsive manner. Cells were treated with different concentrations of WA (upper) or Cel (lower) as shown with or without TNF-α (10 ng/ml) for 24 hours, followed by detection of PARP by Western blotting. Data are shown as mean ±SD of three experiments. (*P<0.05, as compared with the untreated control. #*P*<0.05, WA/Cel treated versus WA/Cel+TNF-α).

To investigate the mechanism of MDA-MB-231 cell death observed in earlier experiments, we performed flow cytometry and Western blotting analysis. The flow cytometric analysis demonstrated that apoptosis was the main mechanism of cell death induced by WA or Cel alone (Fig. 2A), which is consistent with previous reports [2], [4], [5], [6], [7], [14], [15], [16], [17]. TNF-α alone did not caused apoptosis in the MDA-MB-231 cells. TNF-α and WA or Cel combination enhanced (approximately 2-fold) the number of apoptotic cells in a dose-dependent manner compared to each treatment alone (Fig. 2A, B). These results are consistent with the results of MTT assays (Fig. 1A). Furthermore, an increase in cleaved fragments of PARP was detected in cells treated with TNF-α and WA or Cel combination compared to each agent alone (Fig. 2C). These results suggest that TNF-α sensitized MDA-MB-231 cells to WA or Cel resulting in induction of apoptosis.

### TNF-α in combination with WA and Cel induced apoptosis in breast cancer cells by affecting caspase-9 and caspase-3 expression levels

**Figure 3:**
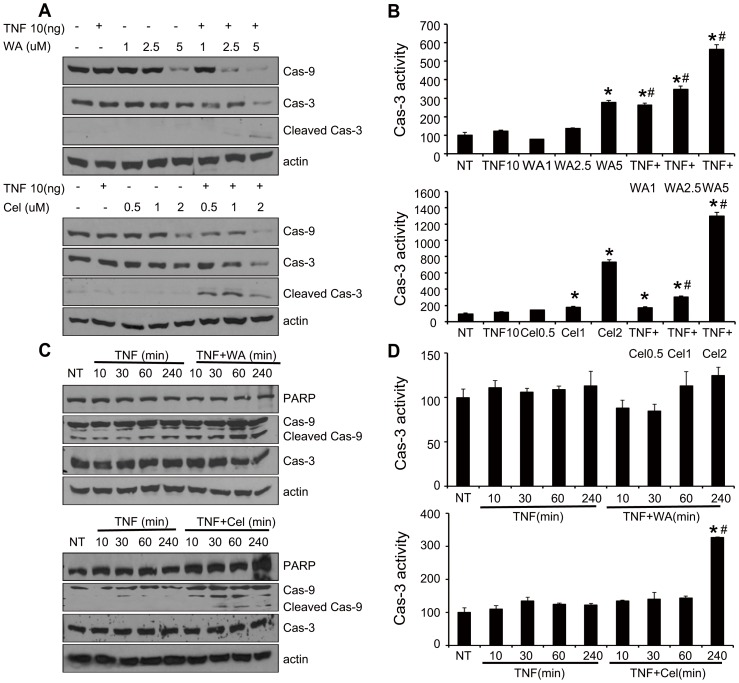
Dose effects of TNF-α plus WA or Cel on caspase-3 and -9 in MDA-MB-231 cells. (A) Concentration dependent effect of WA and TNF-α or Cel and TNF-α combination on caspase-3 and -9 expression. Cells were treated with the indicated concentrations of WA (upper) or Cel (lower) with or without TNF-α (10 ng/ml) for 24 hours, followed by detection of caspase-3 and -9 by Western blotting. (B) Dose effect of WA and TNF-α or Cel and TNF-α combination on caspase-3 activity. Cells were treated with the indicated concentrations of WA (upper) or Cel (lower) with or without TNF-α (10 ng/ml) for 24 hours, followed by detection of caspase-3 activity by adding specific substrate Ac-Asp-Glu-Val-Asp-AMC. (C) Kinetic effects of WA and TNF-α or Cel and TNF-α combination on caspase-3 and -9. Cells were treated with TNF-α (10 ng/ml) or TNF-α (10 ng/ml) plus WA (1 µM) (upper), or TNF-α (10 ng/ml) plus Cel (1 µM) (lower) for the indicated times, followed by detection of caspase-3 and -9 by Western blotting. (D) Kinetic effect of TNF-α plus WA or TNF-α plus Cel on caspase-3 activity. Cells were treated with TNF-α (10 ng/ml) or TNF-α (10 ng/ml) plus WA (1 µM) (upper), or TNF-α (10 ng/ml) plus Cel (1 µM) (lower) for the indicated times, followed by detection of caspase-3 activity via adding the specific substrate Ac-Asp-Glu-Val-Asp-AMC. Data are shown as mean ± SD of three experiments. (*P<0.05, as compared with the untreated control. #*P*<0.05, WA/Cel treated versus WA/Cel+TNF-α).

To deduce the signaling pathways by which TNF-α sensitized MDA-MB-231 breast cancer cells to WA and Cel resulting in apoptosis, cells were treated with WA (1, 2.5, 5 µM) or Cel (0.5, 1, 2 µM) with or without TNF-α and expression levels of caspase-3 and -9, as well as caspase-3 activity, were determined (Fig. 3A, B). TNF-α alone showed no effect on the expression levels of caspase-9 and caspase-3 or caspase-3 activity at 24 hours compared to the DMSO treated group. Although lower doses of WA itself did not affected expression levels of caspase-9, at the dose of 5 µM WA caused reduction in the expression levels of caspase-9. WA had no effect on caspase-3 expression at 24 hours (Fig. 3A, upper), but it increased caspase-3 activity in a dose-dependent manner. The difference between the expression level and activity of caspase-3 might be due to the different sensitivities of Western blotting and specific substrate hydrolysis assays.

Cel showed similar effect to WA on caspase-9 expression levels, although in this case, the effective concentration was 2 µM (Fig. 3A lower). When combined with TNF-α, 2.5 µM WA significantly reduced the expression levels of caspase-9 and caspase-3. It should be noted that we had to use twice the concentration of WA to achieve similar effects when WA was used alone. Cel when combined with TNF-α also reduced caspase-9 expression at the dose of 0.5 µM, only 25% of the effective dose of Cel alone. Cleaved caspase-3 protein levels were dramatically increased in MDA-MB-231 cells treated with combination of TNF-α with WA (2.5, 5 µM) or Cel (0.5, 1, 2 µM) (Fig. 3A). A two-fold increase in caspase-3 activity was observed in MDA-MB-231 cells treated with TNF-α and WA or Cel combination as compared to cells treated with WA or Cel alone. These results are in accordance with the apoptosis detection by flow cytometric analysis (Fig. 2A, B).

To investigate the kinetics of the observed effects, MDA-MB-231 cells were treated with 1 µM WA or Cel with or without TNF-α for 10 to 240 minutes and the expression levels of caspase-3 and caspase-9, as well as caspase-3 activity and PARP cleavage were measured (Fig. 3C, D). As shown in Figure 3C, caspase-9 cleavage increased significantly as early as 10 min after the addition of TNF-α plus WA/Cel, and this effect lasted the full 240 minutes. Following treatment with WA/Cel and TNF-α, no changes in PARP and caspase-3 levels were detected before 240 min (Fig. 3C). In contrast, caspase-9 cleavage and caspase-3 activity gradually increased, reaching a peak at 240 min, following treatment with TNF-α and WA/Cel (Fig. 3C, D). The results indicate that the combination of TNF-α with WA or Cel affected the expression levels of caspase-3 and 9 with an increase in caspase-3 activity in a dose- and time-dependent manner.

### TNF-α did not increase WA- or Cel-mediated proteasome inhibition

Previous studies have shown that the proteasomal β5 subunit is the primary target for WA in vitro and in vivo. WA inhibits the CT-like activity of purified 20S proteasome (IC_50_, 4.5 µM) and 26S proteasome in human prostate cancer cells (5–10 µM) and tumors (4–8 mg/kg), leading to apoptosis induction, angiogenesis suppression, and tumor growth inhibition [6]. Additionally, studies have shown that Cel is a potent inhibitor of the proteasomal CT-like activity in vitro in cultured prostate tumor cells and in vivo in xenograft models. Inhibition of the proteasomal CT-like activity by Cel in prostate cancer cells and xenografts might contribute to its apoptosis-inducing and anti-tumor activities [16].

**Figure 4:**
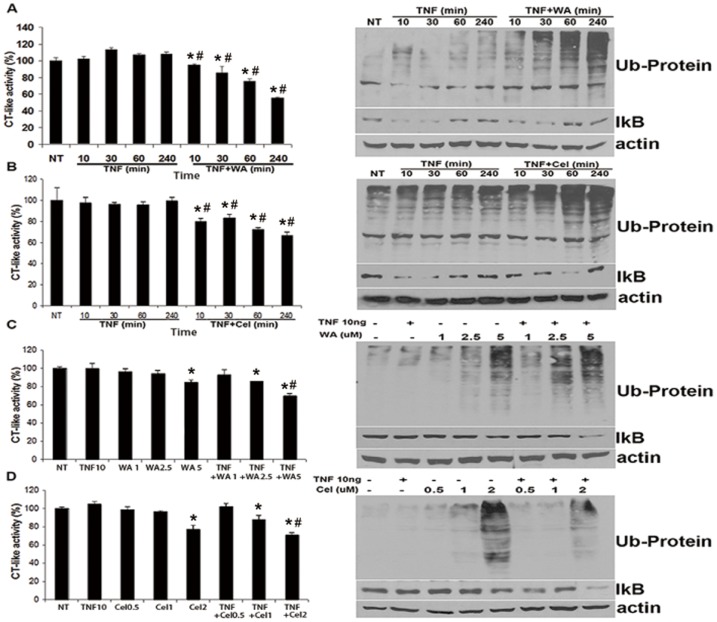
Kinetic and dose effects of TNF plus WA/Cel on cellular proteasome activity in MDA-MB-231 cells. Cells were treated with TNF-α (10 ng/ml) or TNF-α (10 ng/ml) plus WA or Cel for the indicated times and doses, followed by measurement of the proteasomal CT-like activity using Z-GGL-AMC or Western blotting for ubiquitinated proteins and IκBα. (A) Kinetic effects of TNF-α plus WA on CT-like activity, ubiquitinated proteins and IκBα. Cells were treated with WA (2.5 µM) with or without TNF-α for the indicated times. (B) Kinetic effects of TNF-α plus Cel on CT-like activity, ubiquitinated proteins and IκBα. Cells were treated with Cel (1 µM) with or without TNF-α for the indicated times. (C) Dose effects of TNF-α plus WA on proteasomal activity, ubiquitinated proteins and IκBα. Cells were treated with the indicated doses of WA with or without TNF-α for 24 hours. (D) Dose effects of TNF-α plus Cel on proteasomal activity, ubiquitinated proteins and IκBα. Cells were treated with the indicated doses Cel with or without TNF-α for 24 hours. Data are shown as mean ± SD of three experiments. (*P<0.05, as compared with the untreated control. #*P*<0.05, WA/Cel treated versus WA/Cel+TNF-α).

To investigate whether TNF-α sensitized breast cancer cells to WA or Cel by enhancing the inhibition of cellular proteasome activities, we studied the cellular proteasomal CT-like activity and accumulation of ubiquitinated proteins and the proteasome target protein, IκBα. Cells were treated with TNF-α with or without WA or Cel for 240 min. As shown in Figure 4A, TNF-α alone had no significant effect on CT-like activity from 0 min to 240 min. However, inhibition of the CT-like activity was observed in cells treated with TNF-α and WA or Cel combination as compared to TNF-α treatment alone (Fig. 4A, C) in a time-dependent manner. These results are consistent with the enhanced accumulation of ubiquitinated proteins in MDA-MB-231 cells treated with the combination of TNF-α and WA or Cel.

The same parameters were measured in cells treated with varying concentrations of WA (1, 2.5, 5 µM) or Cel (0.5, 1, 2 µM) with or without TNF-α for 24 hours. As shown in Figures 4C and D, TNF-α alone did not have any effect on the proteasomal CT-like activity and accumulation of ubiquitinated proteins compared to DMSO treated cells. WA or Cel inhibited the proteasomal CT-like activity and caused accumulation of ubiquitinated proteins in a dose-dependent manner, which is consistent with previous reports [6], [16]. WA or Cel combined with TNF-α had similar effects, with almost no differences observed in the presence or absence of TNF-α. Taken together, these results indicated that TNF-α did not promote apoptosis of MDA-MB-231 cells by enhancing the inhibitory effects of WA or Cel on proteasomal activity, but by regulating the caspase-3 and caspase-9 components of the apoptotic pathway.

### TNF-α-mediated nuclear translocation of NF-κB was inhibited by WA/Cel

**Figure 5:**
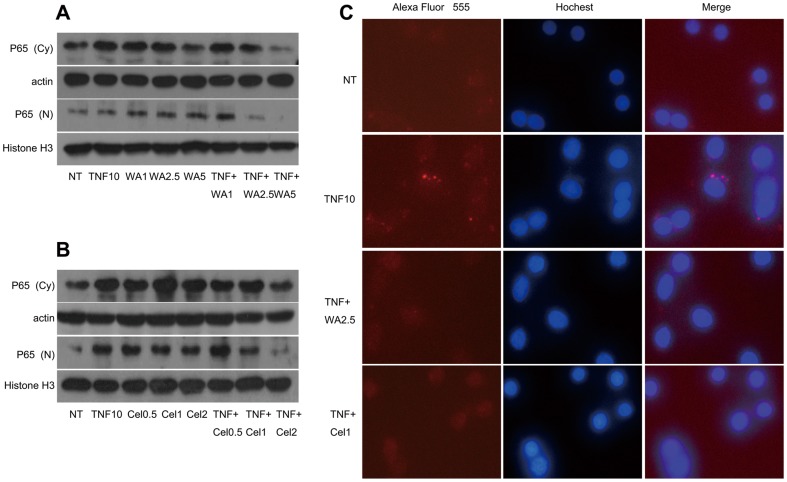
Effect of the combination of TNF-α and WA/Cel on nuclear translocation of NF-κBp65 in cells. (A, B) The effects of WA (A) or Cel (B) on NF-κBp65 activation. MDA-MB-231 cells were treated with different concentrations of WA or Cel with or without TNF-α (10 ng/ml) for 24 hours. The levels of NF-κBp65 in cytoplasmic and nuclear fractions were analyzed by Western blotting. β-actin and Histone H3 were used as the cytoplamsic and nuclear marker respectively. (C) Inhibition of NF-κB binding activity by WA or Cel in TNF-α-stimulated MDA-MB-231cells. Cells were treated with TNF-α (10 ng/ml) with or without WA (2.5 µM) or Cel (1 µM) for 1.5 h. Nuclear translocation of NF-κBp65 was observed by fluorescence microscope. Data are shown as mean ± SD of three experiments.

Nuclear translocation of NF-κBp65 was measured to verify the capacity of WA or Cel to inhibit the activation of NF-κBp65. Cytoplasmic and nuclear NF-κBp65 was analyzed by Western blotting. NF-κBp65 was retained in the cytoplasm when cells were treated with TNF-α plus WA/Cel compared to WA/Cel alone. Furthermore, nuclear NF-κBp65 showed a dramatic decline with TNF-α plus WA/Cel treatment but not with TNF-α treatment alone. NF-κBp65 in the nucleus was also slightly decreased with WA/Cel alone (Fig. 5A, B). The nuclear translocation of NF-κBp65 was also monitored by immunofluorescence microscopy. WA and Cel inhibited the nuclear translocation of NF-κBp65 stimulated by TNF-α at 1.5 hours (Fig. 5C). These findings confirmed that WA/Cel inhibited TNF-α-induced NF-κBp65 activation in MDA-MB-231 cells.

### TNF-α in combination with WA or Cel affects XIAP levels at later time points

**Figure 6:**
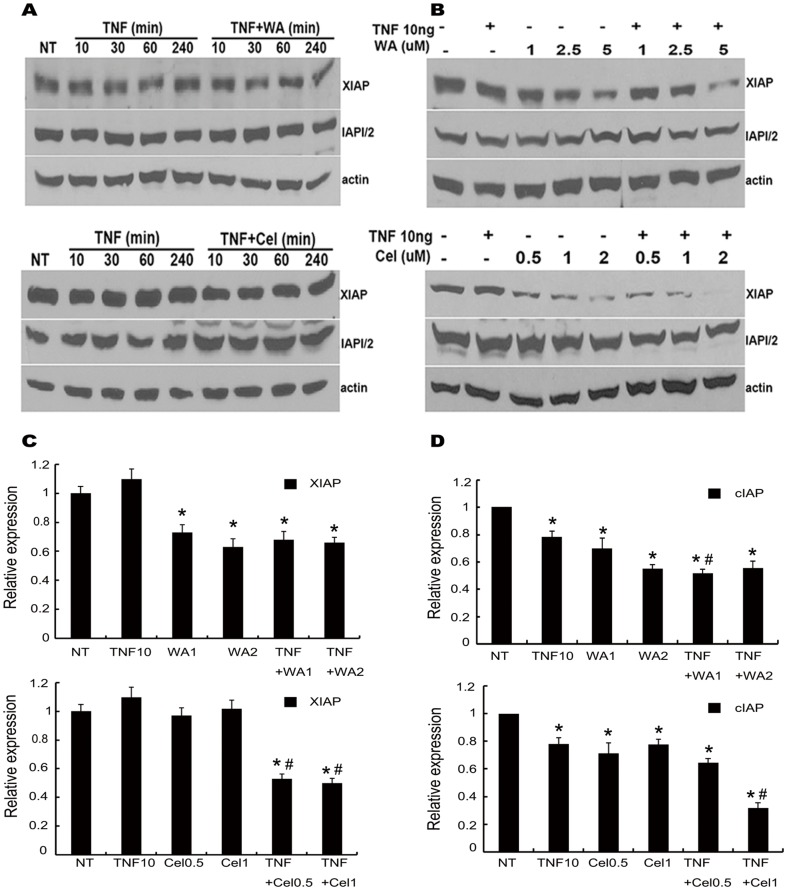
Kinetic and dose effects of TNF and WA/Cel combination on XIAP and IAP1/2 in cells. (A) Kinetic effects of TNF-α plus WA/Cel on XIAP and IAP1/2. Cells were treated with WA (2.5 µM)/Cel (1 µM) with or without TNF-α (10 ng/ml) for the indicated times, followed by Western blotting for XIAP and IAP1/2. (B) Dose effect of TNF-α plus WA/Cel on XIAP and IAP1/2. Cells were treated with the indicated doses of WA/Cel with or without TNF-α (10 ng/ml) for 24 hours, followed by measurement of XIAP, IAP1/2 by Western blotting. (C) Effect of TNF-α plus WA (upper)/Cel (lower) on XIAP mRNA. Cells were treated with WA (1, 2 µM)/Cel (0.5, 1 µM) with or without TNF-α (10 ng/ml) for 12 hours, followed by measurement of XIAP mRNA by RT-qPCR. (D) Effect of TNF-α plus WA (upper)/Cel (lower) on cIAP1/2 mRNA. Cells were treated with WA (1, 2 µM)/Cel (0.5, 1 µM) with or without TNF-α (10 ng/ml) for 12 hours, followed by measurement of cIAP1/2 mRNA by RT-qPCR. Data are shown as mean ± SD of three experiments. (*P<0.05, as compared with the untreated control. #*P*<0.05, WA/Cel treated versus WA/Cel+TNF-α).

The effect of TNF-α in combination with WA or Cel on the expression of IAP family proteins was then investigated by Western blotting (Fig. 6A, B). No reduction in XIAP and cIAP1/2 protein levels were observed between 0 and 240 min following treatment with WA (2.5 µM) or Cel (1 µM) combined with TNF-α (Fig. 6A). As shown in Figure 6B, XIAP protein levels were decreased slightly by treatment with 5 µM WA for 24 hours in the absence of TNF-α, while a marked decrease was observed by when combined with TNF-α. Cel treatment reduced XIAP protein levels in a dose-dependent manner and this effect was markedly enhanced in the presence of TNF-α. The level of cIAP1/2 protein was only slightly decreased after treatment with WA or Cel with or without TNF-α for 24 hours (Fig. 6B).

These results indicated a time-and dose-dependent downregulation of XIAP but not cIAP1/2 protein expression in MDA-MB-231 breast cancer cells treated with WA or Cel combined with TNF-α.

RT-qPCR was performed to investigate the mechanism by which WA or Cel combination with TNF-α caused suppression of XIAP protein expression. Expression of XIAP mRNA showed a slight increase compared with that of the control after treatment with TNF-α alone for 12 hours. The level of XIAP mRNA exhibited a marked decrease after treatment with WA for 12 hours. Cel-mediated suppression of XIAP mRNA expression was not observed. Combined with TNF-α, both WA and Cel markedly decreased XIAP mRNA expression compared with the effects of WA and Cel alone (Fig. 6C). The expression of cIAP1/2 mRNA after treatment with TNF-α was only modestly decreased, while it was significantly reduced when treated with Cel (0.5, 1 µM) and TNF-α for 12 hours compared with the control and monotherapy groups. There were no significant differences in the effects of the different doses of Cel when it was used alone. The expression of cIAP1/2 mRNA decreased when WA was used alone for 12 hours in a dose-dependent manner. The effect of WA at 1 µM, but not 2 µM, was enhanced by TNF-α (Fig. 6D). These observations indicated that WA or Cel combined with TNF-α treatment caused transcriptional repression of XIAP and cIAP1/2 and the expression of cIAP1/2 may be regulated mainly at the translational level.

### NF-κBp65 gene silencing inhibited XIAP and cIAP1/2 expression in MDA-MB-231 cells

**Figure 7:**
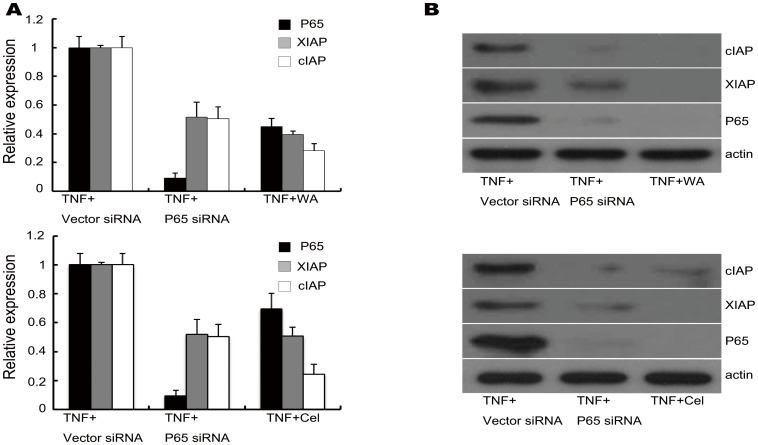
Inhibition of NF-κB target gene expression by WA/Cel and small interfering RNA. (A) MDA-MB-231 cells were transfected with *si*RNA against NF-κBp65 for 6 hours. As a negative control, cells were transfected with the same amount of non targeting control -*si*RNA. Following transfection, cells were treated with TNF-α (10 ng/ml) for 24 hours. Cells that were not transfected were treated with TNF-α (10 ng/ml) plus WA (5 µM) or Cel (2.5 µM) for 24 hours. The mRNA levels of NF-κB-p65, XIAP and cIAP1/2 were detected by RT-qPCR. (B) MDA-MB-231 cells were transfected with *si*RNA against NF-κBp65 for 6 hours. The negative control was treated with the same amount of vector-*si*RNA. Following transfection, cells were treated by TNF-α (10 ng/ml) for 48 hours. Cells that were not transfected were treated with TNF-α (10 ng/ml) plus WA (5 µM) or Cel (2.5 µM) for 48 hours. The levels of NF-κBp65, XIAP and cIAP1/2 proteins were detected by Western blotting. Data are shown as mean ± SD of three experiments.

To further examine whether the expression of NF-κB target genes XIAP and cIAP were affected by NF-κB, expression of NF-κBp65 was knocked down using small interfering RNAs (siRNAs). TNF-α, as a classic NF-κBp65 inducer, was added. After knocking down NF-κBp65, the expression levels of NF-κBp65 mRNA at 24 hours and protein at 48 hours were dramatically reduced by more than 90% compared with that of control siRNA. Knocking down NF-κBp65 significantly reduced XIAP and cIAP gene expression at 24 hours and consistently decreased protein expression at 48 hours. Interestingly, TNF-α plus WA/Cel significantly reduced the gene and protein expression at 24 and 48 hours as well, suggesting that WA/Cel suppressed the expression of NF-κBp65 to interpose the expression of XIAP and cIAP (Fig. 7 A and B).

## Discussion

In this study, we demonstrated that TNF-α could sensitize MDA-MB-231 cells to WA and Cel, leading to apoptosis. One of the major mechanisms by which WA and Cel mediate these effects on breast cancer cells MDA-MB-231 appears to be through suppression of proteasomal activities, resulting in inhibition of IκBα degradation and leading to suppression of NF-κB signaling.

Previous studies have shown that WA and Cel can induce apoptosis in human cancer cells in vitro [4], [5], [6], [7], [15], [16], [17], although the underlying mechanism is poorly understood. In this study, we show that WA and Cel increased the levels of caspase-3,-9 proteins in MDA-MB-231 cells (Fig. 3A), which is consistent with previous reports [37], [38]. Also, our previous studies have shown that WA and Cel inhibit the growth of human prostate cancer cells due to the suppression of proteasomal activities [6], [16]. This study further showed that WA and Cel induce apoptosis in human breast cancer cells through inhibition of the nuclear translocation of NF-κB and downregulation of the expression of XIAP (mRNA and protein) and cIAP1/2 (mRNA), resulting in increased levels of caspase-3 and -9 proteins.

NF-κB is a major key player in TNF-α-mediated biological responses [21], [35], [39]. Members of the IκB family of proteins inhibit NF-κB signaling by binding to and thus sequestering NF-κB in the cytoplasm. Activation of NF-κB signaling is triggered by phosphorylation and ubiquitination of IκBα, leading to its degradation by the ubiquitin-proteasome system, allowing nuclear translocation of NF-κB [40], [41], [42]. In this study, we demonstrate that WA and Cel prevent degradation of IκBα and activation of NF-κB signaling by inhibiting the proteasome, thus abrogating the inhibitory effect of NF-κB signaling on apoptosis.

We further demonstrate that the combination of TNF-α with WA or Cel enhances their individual anti-cancer properties. It has been reported previously that TNF-α induces apoptosis due to its interactions with cell surface receptors such as TNF-α receptor (TNFR) 1 and 2 (extrinsic pathway), and mitochondrial dysfunction (intrinsic pathway) [37], [38], [39]. Binding of specific ligands to cell surface receptors of the TNFR family initiates the extrinsic pathway, while the disruption of cellular integrity by the release of pro-apoptotic factors from the mitochondria triggers the intrinsic pathway of apoptosis. Complex I is formed when TNF-α binds to TNFR-1. This event leads to activation of NF-κB, MAPKs and Complex II, ultimately leading to apoptosis [37], [38], [39]. The members of IAP family of genes are downstream effectors of NF-κB [42], hence, the balance between Complex I and Complex II results in insensitivity to TNF-α-mediated apoptosis. In this study, inhibition of the NF-κB pathway by WA and Cel might have disrupted the balance between Complex I and Complex II, subsequently enhancing the pro-apoptotic effects of TNF-α.

NF-κB, a pro-inflammatory transcription factor, is expressed in almost all cells and tissues. It regulates the expression of more than 500 genes that are involved in various biological events such as cellular transformation, proliferation, survival, invasion, angiogenesis, metastasis and inflammation [38], [39]. The XIAP gene is one of the downstream effectors of NF-κB signaling. When NF-κB signaling is activated, it up-regulates XIAP expression and thereby inhibits apoptosis, leading to evasion of TNF-α-mediated cytotoxicity and drug resistance [42]. Our study found that natural products with proteasomal inhibitory activity suppressed NF-κB activation, resulting in inhibition of XIAP expression, leading to enhanced apoptosis in breast cancer cells. It has been proposed that these natural products could enhance the sensitivity of human breast cancer cells to TNF-α by disturbing the balance between complex I and complex II. WA and Cel, due to their proteasomal inhibitory activities enhance the pro-apoptotic role of TNF-α by inhibiting NF-κB activation. Also, by disturbing the balance between signal transduction pathways that are responsible for insensitivity or resistance to TNF-α signaling, WA and Cel might contribute to inhibition of the local inflammatory response. It can be speculated that this imbalance enhances the sensitivity of human breast cancer cells to the pro-apoptotic effects of TNF-α, ultimately leading to MDA-MB-231 cell death.

Our study is the first to demonstrate that the natural products, WA and Cel, via their proteasomal inhibitory properties, affect the balance between two pathways of TNF-α-mediated apoptosis. We have shown that both WA and Cel reduce activation of NF-κB due to the TNF-α pathway in MDA-MB-231 cells in a similar fashion. The ubiquitin proteasome system is responsible for degradation of the majority of intracellular proteins and thus in turn controls various cellular processes, such as NF-κB activation via the degradation of IκBα [43], [44]. We have shown that WA and Cel inhibit proteasomal activities in MDA-MB-231 cells. It can be speculated that proteasome inhibition mediated by WA and Cel might also contribute to the suppression of the NF-κB pathway. Therefore, it is possible that multiple mechanisms are responsible for enhanced apoptosis in MDA-MB-231 cells when TNF-α is combined with WA or Cel.

A myriad of cytokines are present in the tumor microenvironment that play a crucial role in tumor development. One such cytokine commonly found in the tumor microenvironment is TNF-α. It is involved in inflammation, transplant rejection, septic shock, tumor development and other pathological responses. Various studies indicate that TNF-α has a contradictory role in the tumor development. It promotes apoptosis as well as cell proliferation via regulation of the expression of many cytokines that promote tumor growth. Also, TNF-α levels have been suggested as a potential biomarker for prostate cancer as these are generally higher in tumor tissue than in normal tissue [37], [38], [39]. In accordance with this evidence, our results suggest that TNF-α sensitizes human breast cancer cells, MDA-MB-231 to low doses of WA and Cel, leading to apoptosis due to suppression of the NF-κB signaling pathway. These results underscore the potential of natural proteasome inhibitors as a therapy in certain types of cancer, such as breast cancer, where inflammation plays major role in tumor growth.
